# SALDI Substrate-Based FeNi Magnetic Alloy Nanoparticles for Forensic Analysis of Poisons in Human Serum

**DOI:** 10.3390/molecules27092720

**Published:** 2022-04-23

**Authors:** Sara A. Al-Sayed, Mohamed O. Amin, Entesar Al-Hetlani

**Affiliations:** Department of Chemistry, Faculty of Science, Kuwait University, P.O. Box 5969, Safat 13060, Kuwait; sara.ahmed@grad.ku.edu.kw

**Keywords:** magnetic alloy nanoparticles, pesticides, strychnine, SALDI-MS, forensic analysis

## Abstract

In this study, FeNi magnetic alloy nanoparticles (MANPs) were employed for the forensic analysis of four poisons—dimethametryn, napropamide, thiodicarb, and strychnine—using surface-assisted laser desorption/ionization mass spectrometry (SALDI-MS). FeNi MANPs were prepared via coprecipitation using two reducing agents, sodium borohydride (NaBH_4_) and hydrazine monohydrate (N_2_H_4_·H_2_O), to optimize the prepared MANPs and investigate their effect on the performance of SALDI-MS analysis. Thereafter, SALDI-MS analysis was carried out for the detection of three pesticides and a rodenticide. The prepared substrate offered sensitive detection of the targeted analytes with LOD values of 1 ng/mL, 100 pg/mL, 10 ng/mL, and 200 ng/mL for dimethametryn, napropamide, thiodicarb, and strychnine, respectively. The relative standard deviation (%RSD) values were in the range of 2.30–13.97% for the pesticides and 15–23.81% for strychnine, demonstrating the good spot-to-spot reproducibility of the FeNi substrate. Finally, the MANPs were successfully employed in the analysis of poison-spiked blood serum using a minute quantity of the sample with an LOD of 700 ng/mL dimethametryn and napropamide, 800 ng/mL thiodicarb, and 500 ng/mL strychnine. This study has great potential regarding the analysis of several poisons that may be found in human serum, which is significant in cases of self-harm.

## 1. Introduction 

The World Health Organization (WHO) reported that more than 800,000 people die by suicide every year in low- and middle-income countries. Self-poisoning is considered one of the three most commonly used means to end one’s life [1]. In particular, intentional ingestion of agricultural pesticides in industrial and developing countries has raised serious alarms, owing to their low cost, availability, and fast-acting effect [2,3]. Pesticides account for 4–20% of global suicide rates, causing an approximate death toll of 110,000–168,000 individuals each year [4]. Pesticide poisoning can occur due to occupational, accidental, or intentional exposure; reports show that intentional pesticide poisoning has been responsible for more than 60% of suicide cases in China [5], 71% in Sri Lanka [6], 68% in Trinidad [7], and up to 90% of cases in Malaysia [8]. Strychnine is a rodenticide that is primarily mixed with some drugs, such as heroin [9] and cocaine [10], as an adulterant or diluent. Although it is used in quantities that are not life-threatening, i.e., no more than 2% [9], a slight increase in these quantities may be lethal [11]. Similar to pesticides, strychnine poisoning may occur due to unintended or intended ingestion, and it has been linked to suicidal or homicidal cases [12].

Blood serum, urine, hair, and saliva are often the biological samples of choice to analyze in cases of pesticide poisoning. Blood serum is generally used because it contains the highest concentration of the parent compound rather than the metabolites, and it carries a low risk of contamination [13]. In this respect, several studies have focused on the analysis of pesticides in blood serum; in particular, chromatographic techniques have been utilized to obtain qualitative and quantitative information. Generally, sample extraction and cleanup precede the analysis to eliminate any interferences from the sample matrix. Blood serum is rich in lipids, proteins, sugars, inorganic salts, and pigments, which are likely to interfere with GC-MS analysis [14]. For this reason, several extraction approaches have been proposed, including liquid–liquid extraction (LLE), solid-phase extraction (SPE), and QuEChERS-based methods employed on blood serum prior to LC-MS and GC-MS analyses [15]; on some occasions, several extraction steps are combined [16]. Although useful, these approaches are recognized as complex and laborious, and they consume a large number of chemicals. 

More recently, surface-assisted laser desorption/ionization mass spectrometry (SALDI-MS) has been recognized as a prominent soft ionization technique, which is employed in the analysis of small molecules [17]. It requires minimal sample preparation and produces a clean spectrum with minimal interference from the substrate used. The careful synthesis and tuning of SALDI substrates are essential to obtain maximum performance; the substrate absorbs the laser energy and transfers this energy to the analyte for desorption and ionization, thereby providing minimum fragmentation of the analytes [18]. Several SALDI substrates have been used, such as metal oxide nanoparticles [19], nanocomposites [20], carbon-based materials [21], and others. Due to its advantages, SALDI-MS has been employed in the forensic analysis of drugs [19], biological fluids [22], spiked beverages [23], and other substances.

In this study, we report the first use of FeNi magnetic alloy nanoparticles (MANPs) as a new SALDI-MS substrate for forensic analysis of the poisons dimethametryn, napropamide, thiodicarb, and strychnine. FeNi magnetic alloy nanoparticles (MANPs) were synthesized via the coprecipitation method and then characterized via XPS, BET, UV–Vis spectroscopy, TEM, and VSM. The MANPs showed great SALDI-MS performance and sensitive detection of pesticides and strychnine. The FeNi MANPs were also used in the analysis of the complex samples of human serum spiked with these poisons. 

## 2. Experiments

### 2.1. Chemicals and Reagents 

Iron(II) chloride (FeCl_2_), nickel(II) chloride (NiCl_2_), sodium borohydride (NaBH_4_), hydrazine monohydrate (N_2_H_4_·H_2_O), sodium hydroxide (NaOH), dimethametryn, napropamide, thiodicarb, strychnine, acetonitrile, and ethanol were purchased from Sigma-Aldrich and used without further purification. Deionized water was obtained from an Elix Milli-Q water deionizer and was used in all the experiments. Blood serum samples were purchased from Bio-reclamation, Inc. (Hicksville, NY, USA).

### 2.2. Synthesis of FeNi Magnetic Alloy Nanoparticles

FeNi magnetic alloy nanoparticles (MANPs) were synthesized via a one-pot coprecipitation method using hydrazine monohydrate (N_2_H_4_·H_2_O) or sodium borohydride (NaBH_4_) under a nitrogen atmosphere. Initially, equimolar amounts of FeCl_2_ and NiCl_2_, i.e., 3 mmol of each salt, were dissolved in 150 mL of distilled water and purged with nitrogen for 45 min. Then, 1.00 g of NaOH was dissolved in 25 mL of hydrazine monohydrate and added to the salt mixture dropwise, forming a dark precipitate. The precipitate was allowed to stir and was refluxed at 80 °C for 1 hr under a nitrogen atmosphere. The resultant precipitate was then collected with a magnet and washed several times with water and ethanol and finally dried under vacuum at 85 °C for 24 h. Alternatively, FeNi MANPs were prepared according to the method reported in [24], with some modifications. Equimolar amounts of FeCl_2_ and NiCl_2_ were dissolved in DI water and purged with nitrogen for 45 min. Then, 1 g of NaBH_4_ was added slowly as a powder in excess, forming a dark precipitate which was then stirred and refluxed at 50 °C for 1 h under nitrogen. The black precipitate was collected, washed, and dried as mentioned above. 

### 2.3. Characterization of FeNi Magnetic Alloy Nanoparticles

The surface elemental analysis of the FeNi MANPs was carried out by X-ray photoelectron spectroscopy (XPS) (Thermo Fisher scientific, Waltham, MA, USA); the binding energies were referenced to the C 1s peak at 284.64 eV. The Brunauer–Emmett–Teller (BET) method was used to measure the surface area of the prepared material; this method was applied to the adsorption data by measuring the nitrogen sorption isotherms of the sample at −195 °C using a model Gemini VII, ASAP 2020 automatic Micromeritics sorptometer (Micromeritics, Norcross, GA, USA). UV–Vis spectroscopy was employed to study the optical properties of the obtained material in solid state using Agilent Cary 5000 Scan UV–Vis–near-infrared (UV–Vis–NIR) spectrophotometer (Agilent, Santa Clara, CA, USA). The morphology and particle size of the MANPs were determined using transmission electron microscopy (TEM) employing JEOL JEM 1230 (JEOL Ltd., Akishima, Japan) operated at 120 kV. Measurement of magnetic properties was performed using Lake Shore Model 7410 (Lake Shore, Westerville, OH, USA) vibrating sample magnetometer (VSM) with a moment range between 1 × 10^−7^ emu and 1000 emu at 298 K.

### 2.4. Sample Preparation and SALDI-MS Analysis

**Figure 1:**
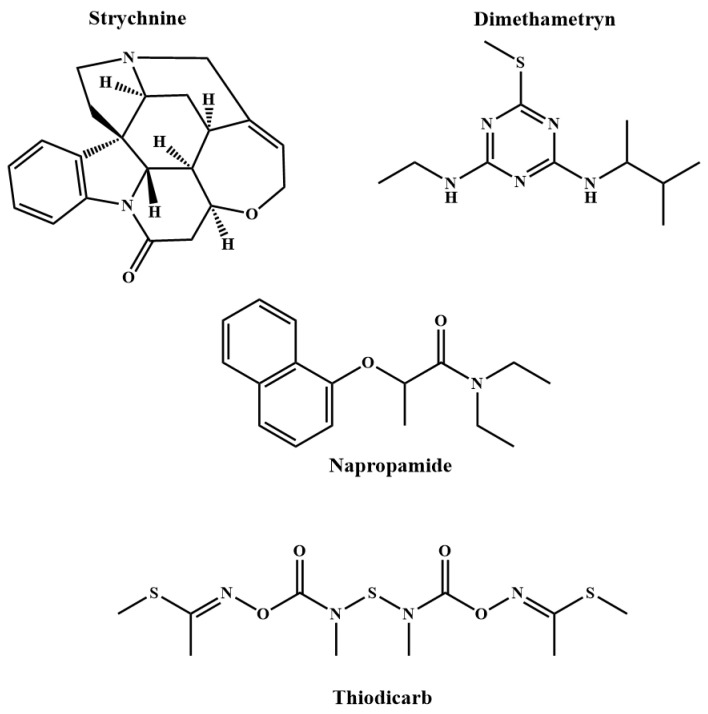
The chemical structures of the studied poisons.

The pesticide standard solution mixture (containing dimethametryn, napropamide, and thiodicarb) and a solution of strychnine were prepared in ethanol at a concentration of 1 mg/mL. Then, 2 µL of each analyte standard solution was mixed with 2 µL of the FeNi MANPs solution (1 mg/mL in ethanol), deposited on the target plate, and allowed to dry at room temperature. The chemical structures for the three pesticides, along with strychnine, are shown in Figure 1.

SALDI-MS analysis was performed using MALDI Bruker ultrafleXtreme MALDI-TOF/TOF-MS system equipped with a Smartbeam-II. The analysis of the pesticide mixture and strychnine was performed in positive ionization mode, and the spectra for the analytes were obtained using a random walk raster with a frequency of 2000 Hz, ion source voltage of 25.0 kV, and reflector voltage of 26.6 kV, over a mass range of 100–500 Da. The instrument was calibrated prior to the analyses using a ProteoMassTM calibrant (Sigma-Aldrich, Chemie GmbH, Schnelldorf, Germany) mixed within the normal range, and the data were processed using Bruker FlexAnalysis (Bruker, Hamburg, Germany).

### 2.5. Analysis of Spiked Human Serum

200 μL Human serum was spiked with 200 μL of different concentrations of the pesticide mixture or strychnine; then, 200 μL of acetonitrile was added to the mixture. The mixture was then vortexed, centrifuged for 5 min at 15,000 rpm, and the supernatant was collected. After that, 2 μL of the supernatant was mixed with 2 μL of FeNi MANPs, deposited on the target plate, and left to dry at room temperature. 

### 2.6. Reproducibility and Limit of Detection (LOD)

The reproducibility of the analyses was determined by obtaining 5–7 spectra for each sample, and the analytes’ average signal intensities and relative standard deviations (%RSD) were computed. For LOD, a range of concentrations of the analytes (1 mg/mL–1 pg/mL) was analyzed, and the LODs were considered at S/N ratio above 3 for the spectral peak.

## 3. Results and Discussion 

### 3.1. Optimization and Characterization of FeNi Magnetic Alloy Nanoparticles

**Figure 2:**
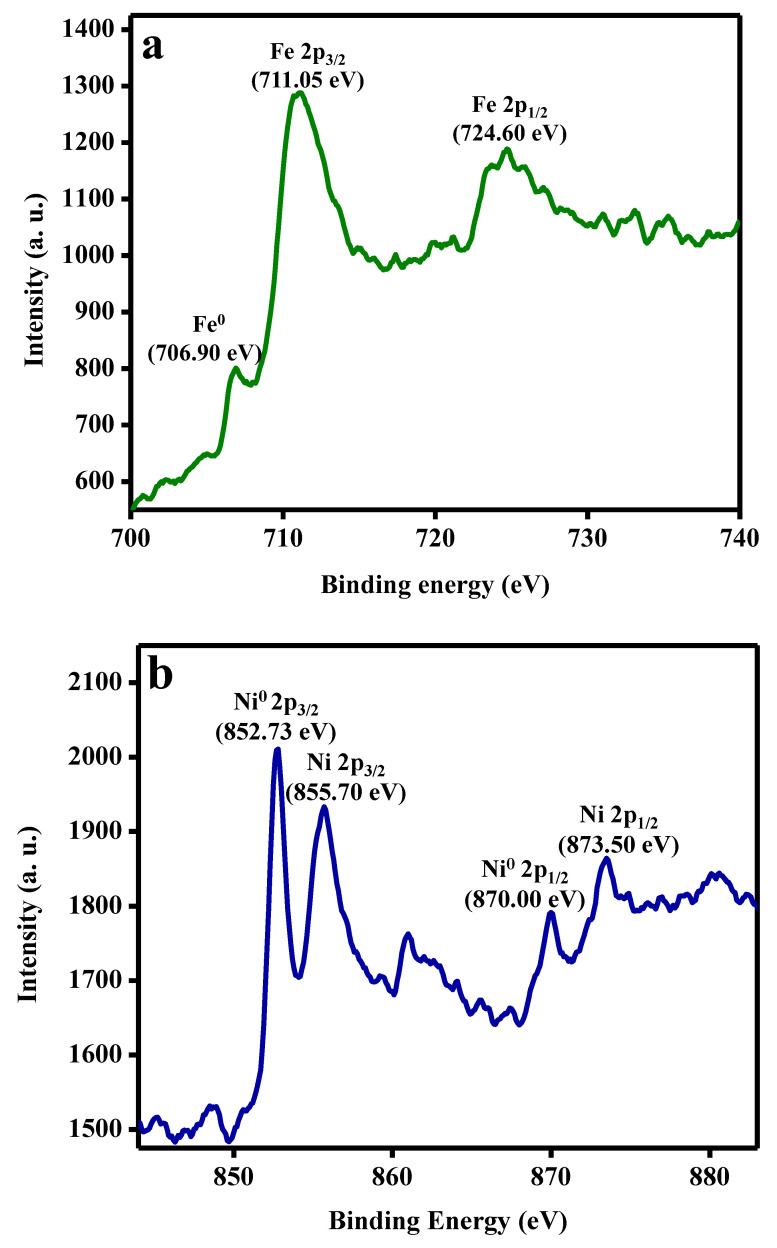

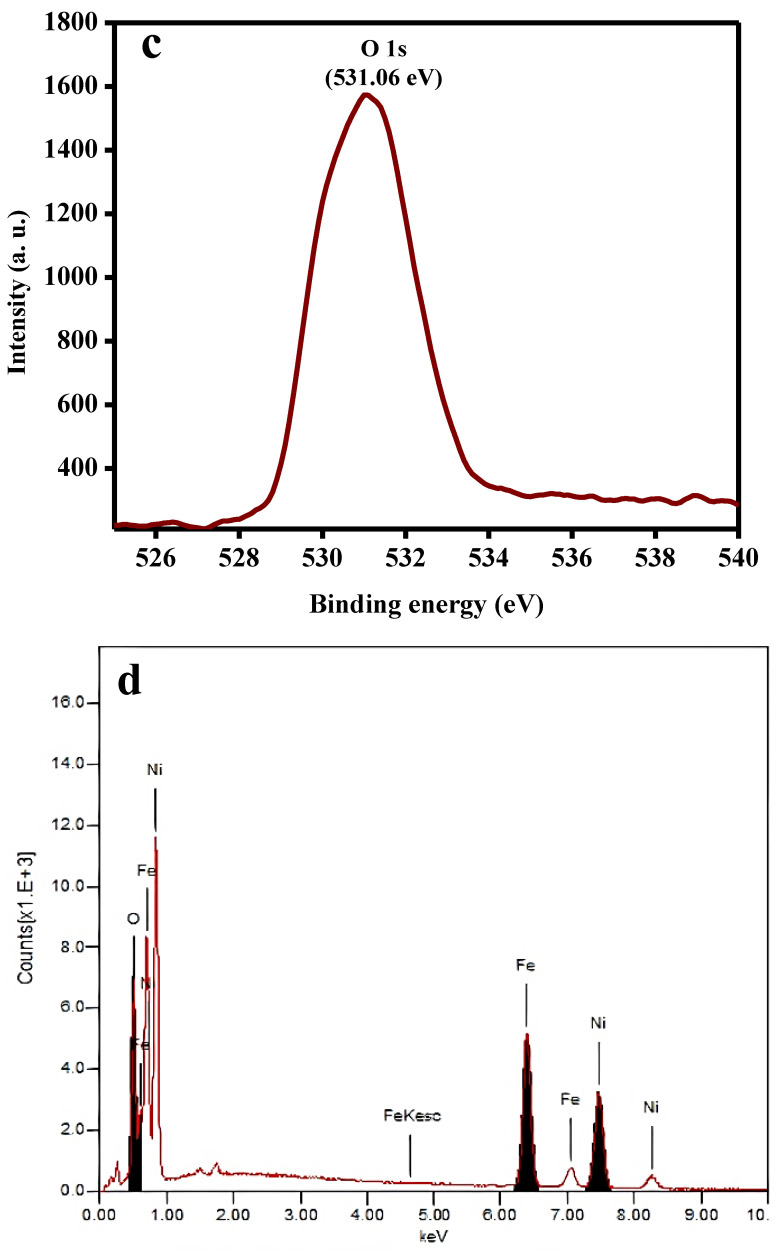
Characterization of FeNi MANPs: (**a**) Fe 2p, (**b**) Ni 2p, and (**c**) O 1s XPS spectra and (**d**) EDS spectrum of FeNi MANPs.

The surface elemental composition and oxidation states of Ni and Fe in the fabricated FeNi MANPs were investigated via X-ray photoelectron spectroscopy (XPS), as shown in Figure 2a–c. Figure 2a illustrates the XPS spectrum of Fe; the binding energy (BE) at 706.90 eV can be ascribed to Fe^0^, and the two peaks at 711.05 and 724.60 eV can be attributed to Fe 2p_3/2_ and Fe 2p_1/2_ of iron oxide, respectively [20,25]. Furthermore, Figure 2b shows a peak centered at BE of 852.73 eV and 870.00 eV, which can be ascribed to Ni 2p_3/2_ and Ni 2p_1/2_ for Ni^0^, and BEs at 855.70 and 873.50 eV, which are attributed to Ni 2p_3/2_ and Ni 2p_1/2_ for Ni^2+^, respectively [26,27]. Finally, a representative O1s spectrum is shown in Figure 2c, showing BE at 531.06 eV, which indicates the presence of oxygen species such as –OH^-^ [28]. These results indicate the successful formation of metallic iron and nickel MANPs, in addition to the hydroxide of both metals on the surface from ambient moisture. Energy dispersive spectroscopy (EDS) measurements were performed on the sample; the EDS analysis of FeNi MANPs is demonstrated in Figure 2d and Table 1. The results confirmed the presence of Fe, Ni, and O, and the ratio of Fe to Ni in the MANPs was almost 1:1.

**Figure 3:**
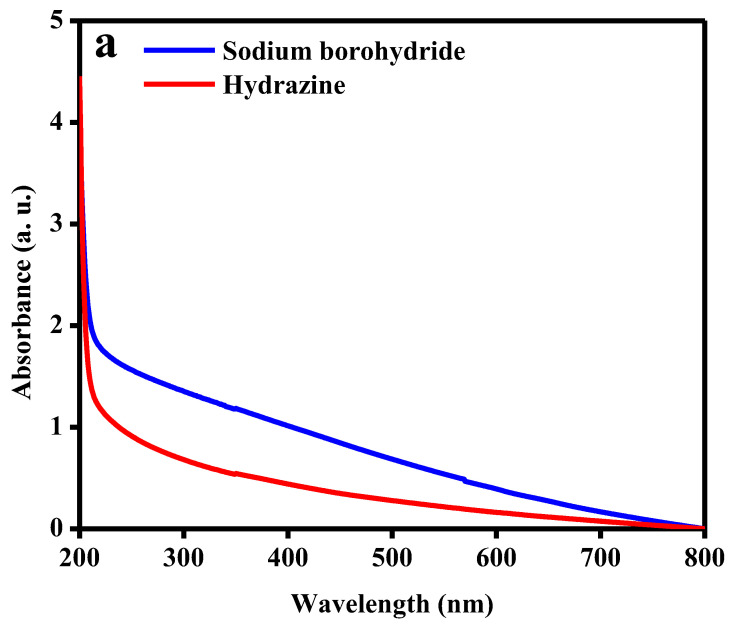

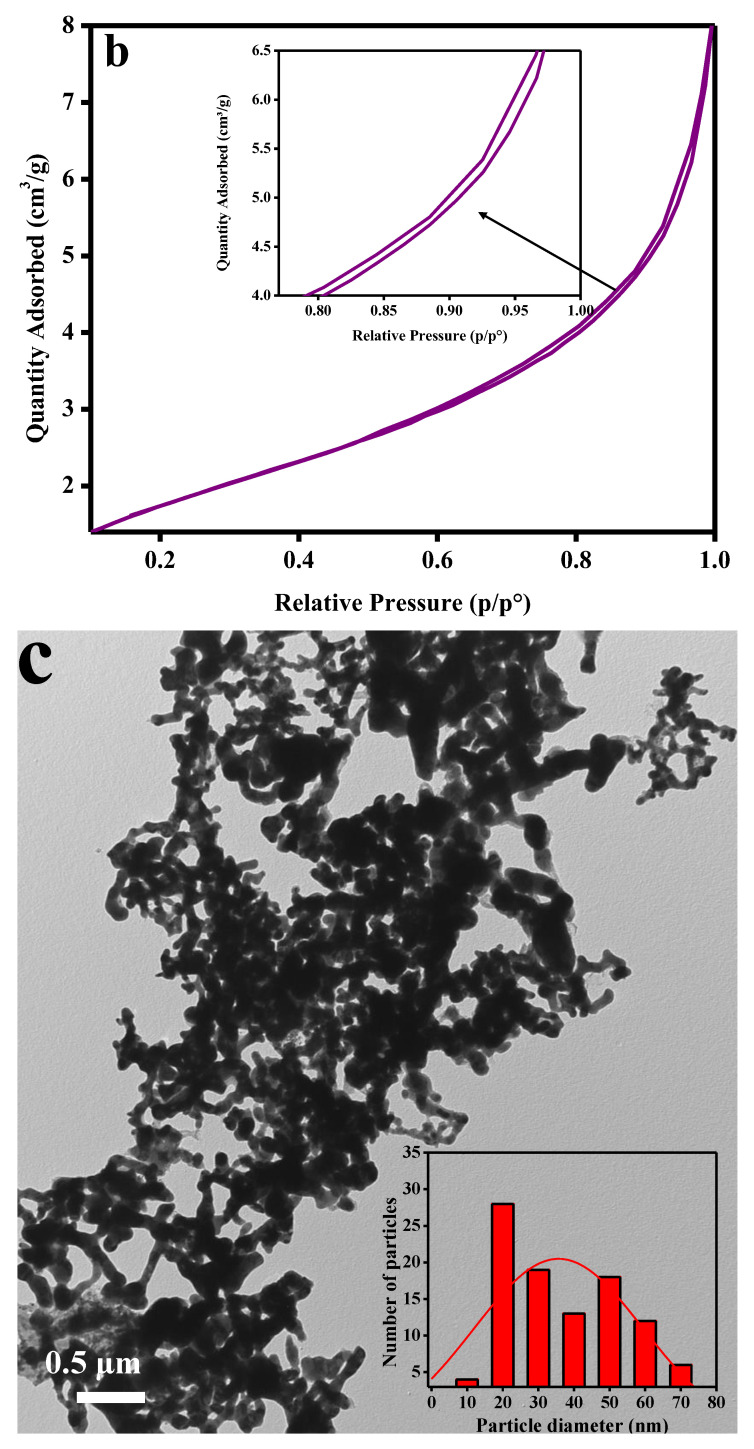

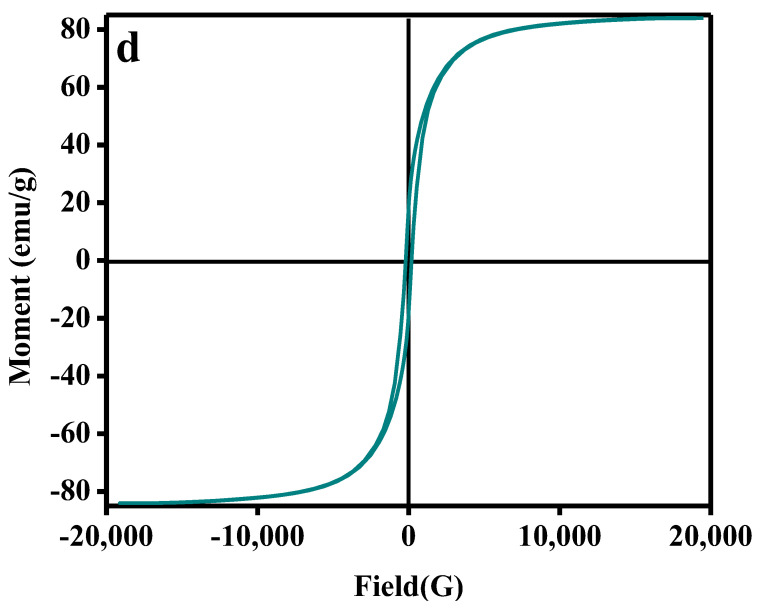
Characterization of FeNi MANPs. (**a**) UV–Vis spectrum; (**b**) BET isotherm (inset shows an enlargement of the hysteresis gap); (**c**) TEM image for the prepared MANPs, and the inset shows particle size distribution; and (**d**) magnetic hysteresis loop of the FeNi MANPs at 298 K.

The efficiency of absorbing the laser irradiation by the substrate and the ability to transfer this energy to the analyte are crucial elements in SALDI analysis [29]. Therefore, nanoparticles with good absorbance in the UV region offer an enhanced SALDI performance and increase the efficiency of analyte detection [30,31]. The UV–Vis spectra of FeNi MANPs prepared using both reducing agents are shown in Figure 3a; the spectra revealed a strong absorbance over the visible and UV regions, which is in agreement with the UV–Vis absorbance spectrum published by Slaton et al. [25]. The results obtained demonstrate the relatively higher absorbance of FeNi MANPs prepared by NaBH_4_ as opposed to N_2_H_4_·H_2_O. Other factors such as the size and porosity of the substrates can also contribute to the desorption/ionization efficacy and SALDI detection [32]. For this purpose, the surface area, pore size, and pore volume for FeNi MANPs were determined utilizing N_2_ adsorption–desorption. Figure 3b depicts the adsorption isotherm, which indicates that the material prepared exhibited IVa isotherm and an H3 hysteresis loop demonstrating the mesoporous nature of the MANPs (the inset shows an enlargement of the hysteresis gap) [33]. The surface area measured was 6.47 m^2^/g, whereas the pore volume was 0.012 cm^3^/g, and the pore size was 7.68 nm. The morphology and average particle size of the synthesized MANPs were obtained using TEM, as illustrated in Figure 3c. The prepared MANPs possessed a spherical shape and formed aggregations with an average particle size of around 42.46 nm, as shown in the inset in Figure 3c. Since small particles offer better desorption/ionization, our findings indicated that the prepared MANPs were good candidates for SALDI analysis [34]. Finally, the magnetic properties of the MANPs were studied by using VSM at 298 K as shown in Figure 3d, and the magnetic hysteresis loop of the FeNi MANPs proved the ferromagnetic nature of the MANPs (non-zero coercivity value) [25]. The saturation magnetization (M_s_) was reached at 84.024 emu/g, and the coercivity (H_c_) was found to be 197.88 G. 

### 3.2. Effects of Reducing Agent on SALDI-MS Analysis 

**Figure 4:**
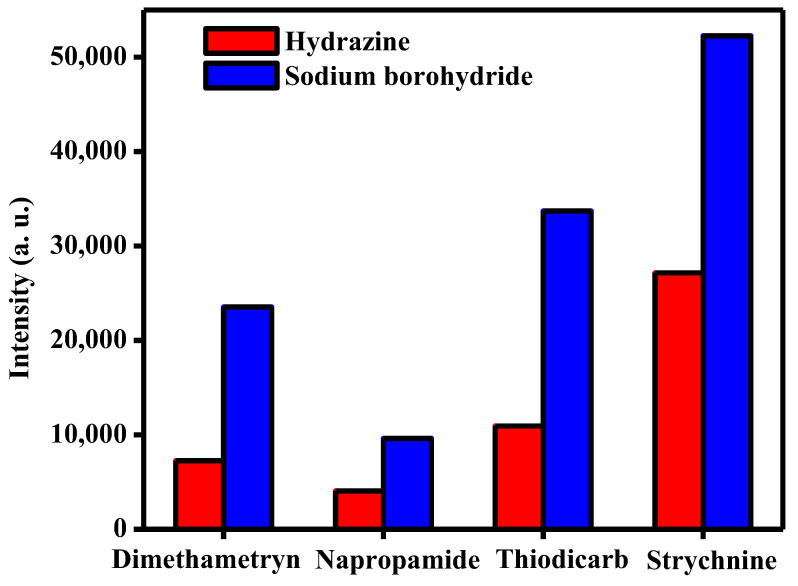
The effect of reducing agents N_2_H_4_·H_2_O and NaBH_4_ on the performance of FeNi MANPs as SALDI-MS substrates for the detection of different poisons: dimethametryn, napropamide, thiodicarb, and strychnine.

In this work, hydrazine monohydrate (N_2_H_4_·H_2_O) and sodium borohydrate (NaBH_4_) were used as reducing agents for the preparation of FeNi MANPs. Therefore, their effect on the performance of SALDI-MS was investigated using the selected poisons by measuring their signal intensities, as shown in Figure 4. The results obtained for dimethametryn, napropamide, thiodicarb, and strychnine indicated that FeNi MANPs prepared using NaBH_4_ produced a greater signal intensity when compared to the ones prepared by N_2_H_4_·H_2_O for all targeted analytes. These results are consistent with the UV–Vis absorbance (Figure 3a), where NaBH_4_ showed a stronger absorbance as opposed to N_2_H_4_·H_2_O when using equivalent amounts of both materials. This can be explained by the thermally-driven ionization mechanism, which involves the adsorption of the analyte on the surface of the substrate, then absorption of laser irradiation by the substrate, and the subsequent transfer of the absorbed energy to the analyte [35]. Consequently, a strong UV absorbance is believed to be one of the main contributors to the enhanced ionization and detection process [36]. As our results showed that FeNi MANPs prepared using NaBH_4_ offered a higher absorption in both the UV and visible range, it was employed for further study. 

### 3.3. Analysis of Poisons Using FeNi MANPs 

**Figure 5:**
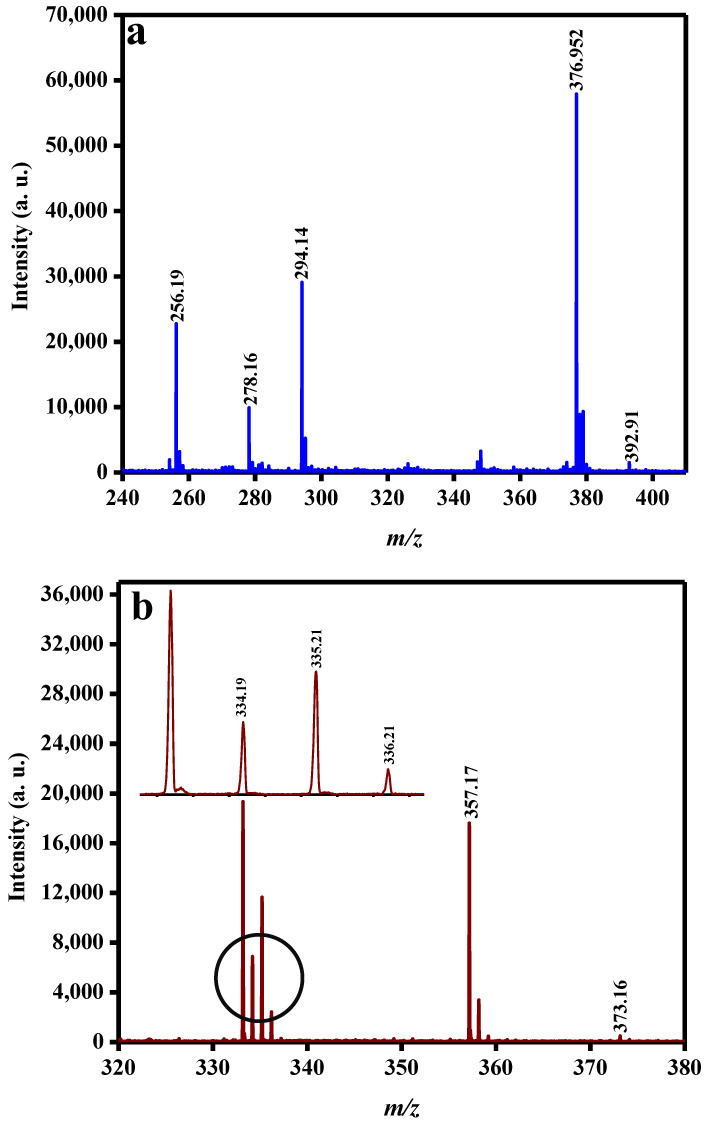
SALDI-MS spectra of (**a**) dimethametryn, napropamide, thiodicarb, and (**b**) strychnine using FeNi MANPs as a substrate.

The analysis of the pesticides and strychnine solutions was performed using SALDI-MS and FeNi MANPs as a substrate; spectra obtained for the three-pesticide mixture and strychnine are shown in Figure 5a,b. The ions obtained, their corresponding *m*/*z*, average intensity, %RSD, and LOD values are displayed in Table 2. Figure 5a depicts the peaks corresponding to the predominant sodiated forms of dimethametryn [Dim + Na]^+^, napropamide [Nap + Na]^+^, and thiodicarb [Thi + Na]^+^, as indicated in the spectra. Additionally, for napropamide, the radical cation [Nap]^+^, protonated [Nap + H]^+^, and potassiated [Nap + K]^+^ forms, along with the protonated form of dimethametryn [Dim + H]^+^ and the potassiated adduct for thiodicarb [Thi + K]^+^, were detected. On the other hand, in Figure 5b, strychnine was obtained in radical ion [Sty]^+^, protonated [Sty + H]^+^, sodiated [Sty + Na]^+^, and potassiated [Sty + K]^+^ forms. The RSD values were 2.30 and 6.16 for [Dim + H]^+^ and [Dim + Na]^+^, respectively, 9.66, 13.97, 8.21, and 11.07 for [Nap]^+^, [Nap + H]^+^, [Nap + Na]^+^, and [Nap + K]^+^, respectively, and 9.27 for [Thi + Na]^+^, whereas the %RSD values for strychnine were 15.83, 17.70, 19.15, and 23.81 for [Sty]^+^, [Sty + H]^+^, [Sty + Na]^+^, and [Sty + K]^+^, respectively. The %RSD values demonstrated the good spot-to-spot repeatability of the SALDI substrate. Finally, LOD values for each targeted analyte were determined, and 1 ng/mL, 100 pg/mL, 10 ng/mL, and 200 ng/mL were obtained for dimethametryn, napropamide, thiodicarb, and strychnine, respectively.

### 3.4. Analysis of Spiked Human Serum Samples

**Figure 6:**
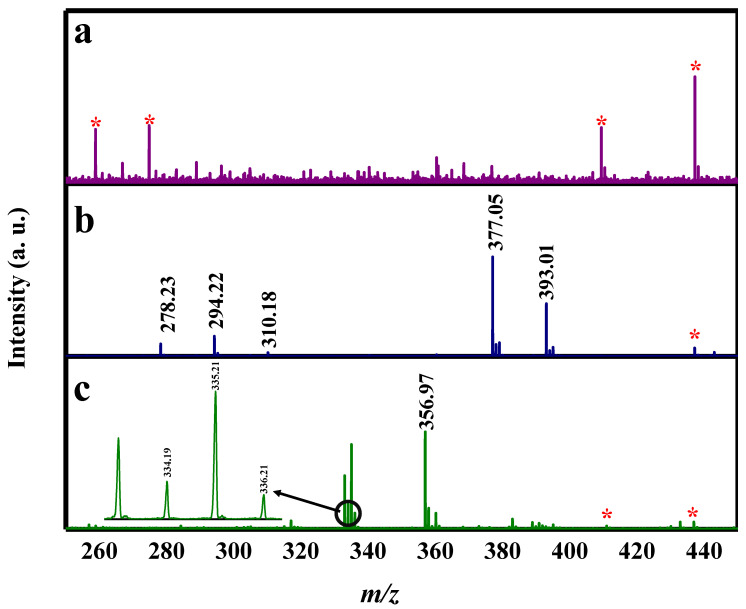
SALDI-MS spectra using FeNi MANPs as substrate. (**a**) control human serum sample. (**b**) dimethamtetryn, napropamide, thiodicarb (the red asterisks point to possible presence of human serum compounds in sample), and (**c**) strychnine in human serum.

Self-poisoning by pesticides has become a common means of suicide in various countries, as well as a source of great concern. The fatality rates for these cases were reported to be as high as 46% in hospital-based studies. The identification and quantification of pesticides in biological samples are considered to be major key evidence in cases of self-poisoning [37]. Additionally, in street-drug culture, the use of rodenticides as diluents is becoming more common at an alarming rate. As recently demonstrated in a study by Blakey et al. [38], the presence of some anticoagulant rodenticides has been identified in some seized 3,4-methylenedioxymethamphetamine (MDMA) tablets as low-level adulterants or contaminants; the risk of being poisoned by such mixed drugs increases with continuous consumption. Herein, FeNi MANPs were employed in the analysis of poison-spiked human serum; dimethametryn, napropamide, thiodicarb, and strychnine in human serum, as well as the LOD for these poisons in human blood serum, were investigated. The initial spectrum for the blank human serum using FeNi MANPs is shown in Figure 6a. Then, pesticide-spiked blood serum spectrum was obtained as demonstrated in Figure 6b; peaks corresponding to the prevalent sodiated forms of dimethametryn [Dim + Na]^+^, napropamide [Nap + Na]^+^, and thiodicarb [Thi + Na]^+^, and potassiated adducts of napropamide [Nap+K]^+^ and thiodicarb [Thi + Na]^+^, were successfully detected. Figure 6c illustrates the spectrum obtained for strychnine in blood serum showing radical [Sty]^+^, protonated [Sty + H]^+^, sodiated [Sty + Na]^+^, and potassiated forms [Sty + K]^+^ of the rodenticide. The ions detected, their corresponding *m*/*z*, their average intensity, %RSD, and their LOD values are displayed in Table 3. The LOD values for the poison-spiked human serum were found to be 700 ng/mL for both dimethametryn and napropamide, 800 ng/mL for thiodicarb, and 500 ng/mL for strychnine. The %RSD values were 22.45% for [Dim + Na]^+^, 14.94 and 18.06% for [Nap + Na]^+^ and [Nap + K]^+^, respectively, and 23.93 and 17.68% for [Thi + Na]^+^ and [Thi + K]^+^, respectively. The RSD values for strychnine were 13.62, 4.65, 2.19, and 9.16% for [Sty]^+^, [Sty + H]^+^, [Sty + Na]^+^, and [Sty + K]^+^, respectively. These values show the good repeatability of the FeNi MANPs as a SALDI substrate, even in a complex sample matrix of human serum. It is worth mentioning that the obtained LODs were 1, 10, and 200 ng/mL for dimethametryn, thiodicarb, and strychnine, respectively, and 100 pg/mL for napropamide in human serum. The achieved values are below the lethal dose values for all the poisons; the reported values for dimethametryn, napropamide, thiodicarb, and strychnine are higher than 3000, 4680, 50, and between 1 and 2 ppm, respectively [39,40].

A few studies have focused on the laser desorption ionization (LDI) analysis of human serum using nanoparticles, nanocomposite, inorganic monoliths, and others, as summarized in Table 4. For instance, FePtCu-SO_3_ NPs were applied for the detection of lysozyme in human serum [41], while silicon nanopost arrays were employed for the analysis of small metabolites and lipids in human serum [42]. Silver NPs were also applied for the analysis of mouse blood serum to detect glucose and the anti-cancer drug 5-fluorouracil with a good mass accuracy of 0.007 for the drug [43]. Additionally, our group has previously studied the detection of pesticides in human serum samples using copper ferrite NPs [44] and modified silica monolith [23] as SALDI substrates. Compared with the current study, the results obtained in this work are satisfactory; however, the LOD values obtained using FeNi MANPs were slightly higher than the results obtained using SALDI-MS with other substrates, which could be due to the limited surface area of the metallic substrate. Thus, future work may involve the modification of FeNi MANPs with carbon-based material, noble metals, or a metal–organic framework (MOF) to increase the surface area and, consequently, SADLI analysis. 

## 4. Conclusions

FeNi magnetic alloy nanoparticles (MANPs) were synthesized using sodium borohydride (NaBH_4_) and hydrazine monohydrate (N_2_H_4_·H_2_O) as reducing agents. The prepared MANPs were characterized using a range of analytical techniques to investigate their chemical and morphological properties. The prepared MANPs using sodium borohydride (NaBH_4_) showed enhanced detection of poisons in comparison to hydrazine monohydrate (N_2_H_4_·H_2_O). The choice of reducing agent influenced the prepared materials, producing MANPs of good absorbance in the visible and UV regions, thus exhibiting great performance as a SALDI-MS substrate. Three pesticides and a rodenticide were analyzed utilizing FeNi MANPs as a SALDI-MS substrate. The (LOD) values for dimethametryn, napropamide, thiodicarb, and strychnine were found to be 1 ng/mL, 100 pg/mL, 10 ng/mL, and 200 ng/mL, respectively. The MANPs were also used in the analysis of poison-spiked human serum, with LOD values of 700 ng/mL for dimethametryn and napropamide, 800 ng/mL for thiodicarb, and 500 ng/mL for strychnine. Further chemical modification of the prepared MANPs needs to be performed to enhance their surface area and SALDI-MS performance.

## Figures and Tables

**Table 1:** Mass (%) and Atom (%) for FeNi using EDS.

Element	Mass (%)	Atom (%)
Ni	48.13	37.04
Fe	41.44	33.53
O	10.42	29.43

**Table 2:** Poisons detected, ion formed, *m*/*z*, average intensity, %RSD, and LOD.

Compound Name	Ion Formed	*m*/*z*	Average Intensity	%RSD	LOD
Dimethametryn	[Dim + H]^+^	256.19	23,077.00	2.30	1 ng/mL
	[Dim + Na]^+^	278.18	11,366.61	6.16	
Napropamide	[Nap]^+^	271.18	735.47	9.66	100 pg/mL
	[Nap + H]^+^	278.16	660.36	13.97	
	[Nap + Na]^+^	294.14	28,464.64	8.21	
	[Nap + K]^+^	310.08	1204.82	11.07	
Thiodicarb	[Thi + Na]^+^	376.95	76,877.32	9.27	10 ng/mL
Strychnine	[Sty]^+^	334.28	11,014.64	15.83	200 ng/mL
	[Sty + H]^+^	335.29	16,570.42	17.70	
	[Sty + Na]^+^	357.26	34,571.31	19.15	
	[Sty + K]^+^	373.25	1838.68	23.81	

**Table 3:** Poisons detected, ion formed, *m*/*z*, average intensity, %RSD, and LOD in human serum.

Compound Name	Ion Formed	*m*/*z*	Average Intensity	%RSD	LOD (ng/mL)
Dimethamtetryn	[Dim + Na]^+^	278.23	2582.67	22.45	700
Napropamide	[Nap + Na]^+^	294.22	5649.92	14.94	700
	[Nap + K]^+^	310.18	1089.33	18.06	
Thiodicarb	[Thi + Na]^+^	376.05	20,357.78	23.93	800
	[Thi +K]^+^	393.01	7831.50	17.68	
Strychnine	[Sty]^+^	334.19	608.68	13.62	500
	[Sty + H]^+^	335.21	659.65	4.65	
	[Sty + Na]^+^	356.97	21,779.79	2.19	
	[Sty + K]^+^	373.06	2430.85	9.16	

**Table 4:** Comparison of the findings of previous investigations and our study by substrate used, analyte, and LOD.

Substrate Used	Surface Area (m^2^/g)	Analyte	LOD	Ref
Silicon nanopost arrays	-	Small metabolites and lipids	-	[42]
Silver NPs	-	5-fluorouracil	-	[43]
FePtCu NPs	-	Lysozyme	-	[41]
Au-SiO_2_ monolith	368.2	Dimethametryn and thiodicarb	100 ng/mL	[23]
		Napropamide and metalaxyl	1 ng/mL	
CuFe_2_O_4_ NPs	19.7	Napropamide	10 μg/mL	[44]
		Metalaxyl	10 ng/mL	
		Thiodicarb	100 pg/mL	
FeNi NPs	6.47	Dimethametryn	700 ng/mL	This work
		Napropamide	700 ng/mL	
		Thiodicarb	800 ng/mL	
		Strychnine	500 ng/mL	

## Data Availability

Not applicable.

## Sample Availability

Samples of the compounds are not available from the authors.
